# Intravenous Thrombolysis is Effective in Young Adults: Results from the Baden-Wuerttemberg Stroke Registry

**DOI:** 10.3389/fneur.2015.00229

**Published:** 2015-11-04

**Authors:** Björn Reuter, Christoph Gumbinger, Tamara Sauer, Horst Wiethölter, Ingo Bruder, Curt Diehm, Peter A. Ringleb, Rolf Kern, Werner Hacke, Michael G. Hennerici, Stroke Working Group of Baden-Wuerttemberg

**Affiliations:** ^1^Department of Neurology and Neurophysiology, University Hospital Freiburg, Freiburg, Germany; ^2^Department of Neurology, Universitätsmedizin Mannheim, Heidelberg University, Mannheim, Germany; ^3^Department of Neurology, University Hospital Heidelberg, Heidelberg, Germany; ^4^Formerly affiliated with Department of Neurology, Bürgerhospital Stuttgart, Stuttgart, Germany; ^5^Office for Quality Assurance in Hospitals (GeQiK), Baden-Wuerttembergische Hospital Association, Stuttgart, Germany; ^6^Department of Internal/Vascular Medicine, Max-Grundig-Klinik, Bühl, Germany; ^7^Department of Neurology, Klinikum Kempten, Kempten, Germany; ^8^Full list of contributors can be found at the end of the article

**Keywords:** acute stroke, IV thrombolysis, age, young adults, epidemiology of stroke, stroke unit concept

## Abstract

**Background:**

The efficacy of intravenous thrombolysis (IVT) is sufficiently proven in ischemic stroke patients of middle and older age by means of randomized controlled trials and large observational studies. However, data in young stroke patients ≤50 years are still scarce. In this study, we aimed to evaluate the effectiveness and safety of IVT in young adults aged 18–50 years. Data from a consecutive and prospective stroke registry was analyzed that covers a federal state with 10.8 million inhabitants in southwest Germany.

**Methods:**

Our analysis comprises 51,735 ischemic stroke patients aged 18–80 years and hospitalized from January 2008 to December 2012. Of these, 4,140 (8%) were aged 18–50 years and 7,529 (15%) underwent IVT. Data on 8,439 patients (16% of the study population) were missing for National Institutes of Health stroke severity score at admission and/or modified Rankin Scale (mRS) at discharge and were excluded from outcome analysis. In sensitivity analysis, patients with incomplete data were also examined. Binary logistic regression models were used adjusted for patient, hospital, and procedural parameters and stratified by age group (18–50 and 51–80 years, subgroup analyses 18–30, 31–40, and 41–50 years) to assess the relationship between IVT and mRS at discharge.

**Results:**

IVT appears equally effective in young adults 18–50 years (adjusted odds ratio 1.40, 95% confidence interval 1.12–1.75; *p* = 0.003), compared to patients 51–80 years of age (1.33, 1.23–1.43; *p* < 0.001). Age-stratified analyses suggest an inverse relation of age and effectiveness, which appears to be highest in very young patients 18–30 years of age (2.78, 1.10–7.05; *p* = 0.03).

**Discussion:**

Ischemic stroke etiology, vascular dynamics, and recovery in young patients differ from those of middle and older age. The evidence from routine hospital care in Germany indicates that IVT in young stroke patients appears to be at least equally effective as in the elderly.

## Introduction

Despite very noticeable advances in acute stroke treatment, rehabilitation therapy and secondary stroke prevention, stroke still remains as one of the leading causes of death and disability. The progressively increasing life-expectancy in high-income societies was expected to be the main reason for a higher incidence of stroke. However, the recently published global burden of disease study demonstrated an increasing incidence of stroke in young adults (1). These data finally confirm the observations made from regional or national stroke registries in Scandinavia and northern America (2–6). The Baden-Wuerttemberg (BW) stroke registry covers a federal state with 10.8 million inhabitants and collects data of stroke patients >18 years of age and admitted to hospitals within 7 days of stroke onset. In line with other reports, between 2007 and 2011, a constant increase of hospitalized stroke patients 18–59 years of age from 4,040 in 2007 (13.8% of the annual cohort) to 5,097 (14.3%) in 2011 was observed (7).

Besides common cardiovascular risk factors, several other stroke etiologies have to be taken into account in young adults. This comprises drug intake, lifestyle risk factors, traumatic and inflammatory vascular disease, hereditary cerebrovascular malformations, migraine, cardiac disease, and several genetic disorders of blood cell lines, blood coagulation, connective tissue, cerebral white matter, lysosomal storage diseases, and mitochondria (8–10).

Acute ischemic stroke treatment in young patients should be performed equally to patients of middle and older age (11). However, while the efficacy and effectiveness of intravenous thrombolysis (IVT) with rt-PA are sufficiently proven in ischemic stroke patients of middle and older age by means of randomized controlled trials and large observational studies, data on young ischemic stroke patients from large cohorts are still limited. Data from the BW stroke registry were used stratified by age to provide further evidence on IVT in young adults.

## Materials and Methods

We conducted a retrospective observational study based on a large and consecutive hospital-based stroke registry. The study was approved by the ethics committee of the Medical Faculty, University of Heidelberg (S339-2012) and by the governing board of the GeQiK (12).

### Setting

Baden-Wuerttemberg has more than 10 million inhabitants and approximately 140 hospitals involved in acute stroke care. In 1998, BW implemented a structured three-level medical concept for the treatment of stroke [for detailed information see in Ref. (7)]. Since 2004, this concept is monitored with a consecutive and prospective stroke database. Participation is mandatory for all hospitals involved in acute stroke care. Therefore, the database represents the broad spectrum of hospitals with stroke care under responsibility of departments of internal medicine and neurology, consisting of general wards, stroke units, and intensive care units. Data covering a period of 5 years, from January 1, 2008 to December 31, 2012 were analyzed in the present study. The cohort was divided into age-stratified subgroups (18–50 and 51–80 years, further subgroup analyses 18–30, 31–40, and 41–50 years, respectively).

### Eligibility Criteria and Study Size

**Figure 1:**
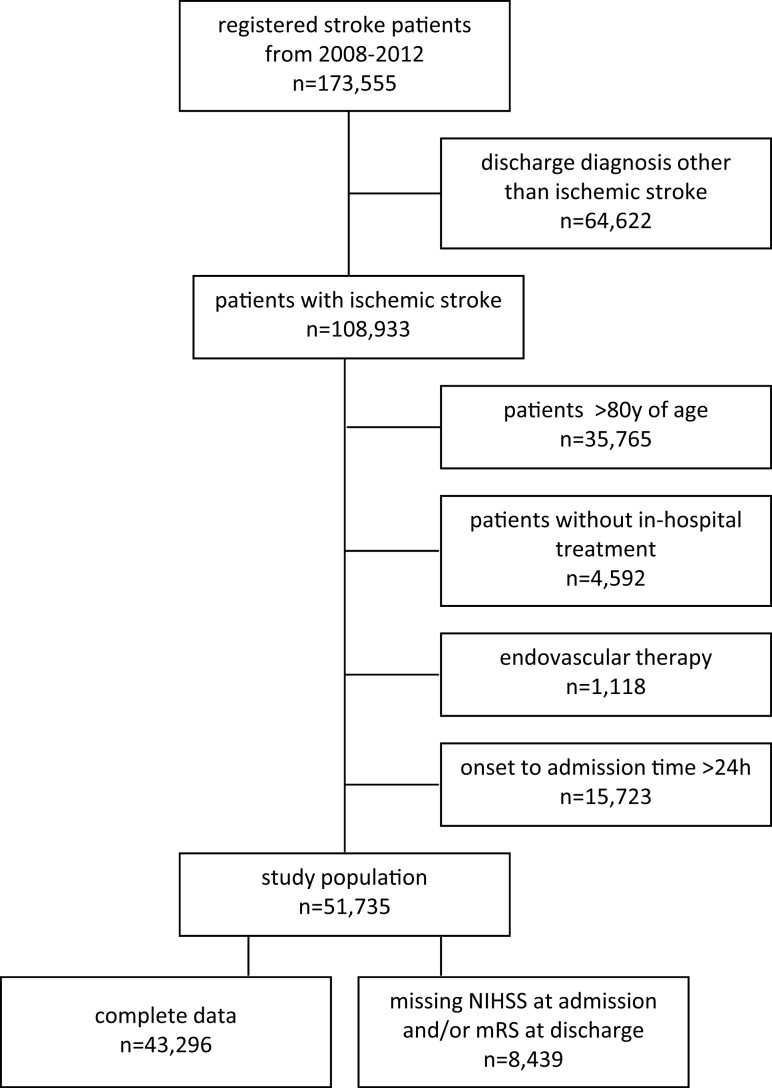
**Study cohort selection flow diagram**.

From January 2008 to December 2012, 173,555 patients were hospitalized in BW for having suffered an ischemic stroke, intracerebral hemorrhage, or transient ischemic attack within 7 days of onset. Of those, 108,933 were discharged with ICD10 diagnosis of ischemic stroke (Figure 1). Patients aged >80 years, with admission for diagnosis of ischemic stroke but without in-patient treatment, patients who underwent endovascular therapy, and patients with onset to admission time of more than 24 h were excluded from our analysis, in the latter to avoid a bias due to early spontaneous recovery in the non-treatment group. Patient characteristics and procedural parameters of the remaining 51,735 patients are described in detail and the effect of IV thrombolysis on disability is assessed. Of those, 8,439 patients were initially not included into our outcome analysis because of missing National Institutes of Health stroke scale (NIHSS) scores at admission (*n* = 6,245, 12.1% of the study population) and/or modified Rankin Scale (mRS) scores at discharge (*n* = 2,438, 4.7% of the study population). Missing documentation of adjustment or endpoint variables was 13% (*n* = 539) and 16.6% (*n* = 7,900) in patients aged 18–50 and 51–80 years, respectively. This left 43,296 patients suitable for our primary statistical analysis. A secondary sensitivity analysis for all 51,735 patients was conducted by imputation of missing variables to identify any substantial differences.

### Variables

Documentation includes patient demographic data, medical history, hospital admission time, level of hospital care, admitting department and ward, nature and timing of diagnostic procedures, IVT, in-hospital complications, discharge information, and hospital mortality. Stroke severity at admission was assessed using the NIHSS and mRS score. A pre-stroke mRS score was documented at admission to estimate acute deterioration of functional ability. At discharge, a final mRS score was assessed.

The binary outcome variables “mRS ≤ 1 or not worse than pre-stroke mRS” and mortality were considered useful to measure the clinical effectiveness of IVT. This mRS outcome parameter has delivered an optimal performance in clinical practice because a relevant proportion of patients with a pre-stroke mRS ≥ 2 would otherwise *per se* not achieve a favorable outcome.

### Statistical Analysis

We first used standard descriptive statistics to explore differences in patient characteristics, stroke care, and clinical outcomes across the age groups. Multiple logistic regression models were then used to assess the association between IVT and binary clinical outcomes. The models were fitted both stratified and unstratified by the age group and were adjusted for patient characteristics (pre-stroke and admission mRS score, NIHSS, prior stroke event, diabetes, atrial fibrillation), admitting facility, and length of hospital stay. For unstratified analyses of the total study cohort, adjustment was also performed for age.

Sensitivity analyses were conducted with imputed values in case of missing NIHSS ad admission and mRS at discharge. For the NIHSS, authors imputed the median age- and treatment group-specific score and for the mRS at discharge the mRS at admission was carried forward. This strategy was deemed conservative by the authors as it implies no treatment effect for observations with incomplete data.

All statistical tests were two-sided, and *p* values of <0.05 were considered to be statistically significant. The analyses were carried out using SAS 9.3 (SAS Institute Inc., Cary, NC, USA).

## Results

### Patient Baseline Characteristics

Patient characteristics are shown in Table 1. Out of 51,735 ischemic stroke patients, 4,140 (8%) were aged 18–50 years and 47,595 (92%) 51–80 years. In further age-stratified subgrouping, 320 (1%) of the young adults were 18–30 years, 780 (2%) 31–40 years, and 3,040 (6%) 41–50 years of age. The gender ratio was equally balanced between the stroke patients aged 18–50 and 51–80 years with approximately 40% being females. Within the age cohort 18–50 years, the proportion of females was highest in very young adults 18–30 years (57%) and then showed a constant decline with increasing age. As expected, the estimated pre-stroke mRS and the frequency of comorbidities were more favorable in young adults, who also presented with lower median NIHSS indicating less severe stroke symptoms. The percentage of patients previously having suffered an ischemic stroke was 12% in young adults and 25% in adults of middle and older age.

**Table 1:** **Patient characteristics**.

Variable	Age group (years)				
	18–50	51–80	18–30	31–40	41–50
Patients, *n* (%)	4,140 (8)	47,595 (92)	320 (1)	780 (2)	3,040 (6)
Female sex, *n* (%)	1,659 (40)	19,394 (41)	181 (57)	347 (45)	1,131 (37)
Pre-stroke mRS score, *n* (%)					
0	3,682 (89)	32,293 (68)	302 (94)	712 (91)	2,668 (88)
1	239 (6)	5,940 (13)	10 (3)	41 (5)	188 (6)
2	125 (3)	4,686 (10)	4 (1)	19 (2)	102 (3)
3	70 (2)	3,085 (7)	3 (1)	6 (1)	61 (2)
4	22 (1)	1,292 (3)	1 (0)	2 (0)	19 (1)
5	2 (0)	299 (1)	0	0	2 (0)
NIHSS, median (IQR)	3 (1, 6)	4 (2, 8)	2 (1, 5)	2 (1, 6)	3 (1, 6)
Missing NIHSS, *n* (%)	385 (9)	5,860 (12)	32 (10)	73 (9)	280 (9)
Comorbidities, *n* (%)					
Arterial hypertension^a^	1,123 (27)	24,803 (84)	22 (10)	131 (27)	970 (51)
Hypercholesterolemia^a^	910 (22)	16,255 (55)	29 (13)	111 (23)	770 (40)
Atrial fibrillation	167 (4)	11,697 (25)	5 (2)	22 (3)	140 (5)
Diabetes mellitus	407 (10)	14,001 (29)	9 (3)	52 (7)	346 (11)
Prior stroke event, *n* (%)	489 (12)	12,109 (25)	24 (8)	72 (9)	393 (13)

*^a^Information was not routinely documented over the entire study period and is therefore missing for N = 19,703 patients*.

*IQR, interquartile range; mRS, modified Rankin Scale; NIHSS, National Institutes of Health stroke scale*.

Further stratification according to age and IVT demonstrated several differences between the treatment group and controls (Table S1 in Supplementary Material). Patients receiving IVT were previously in a better functional state according to the estimated pre-stroke mRS score and, in line with this finding, had less frequently suffered a previous ischemic or hemorrhagic stroke. However, they presented with more severe stroke symptoms assessed with the NIHSS score at admission. These observations were independent from age.

### Procedural Parameters

Procedural parameters are presented in Table 2. Young adults were more frequently admitted to hospitals providing maximum care, with the highest proportion observed in patients aged 18–30 years. Chances of being treated in specialized stroke units were almost independent from age. IVT rates were 14% in patients aged 51–80 years and 18% in patients 18–50 years. No difference within the further age-stratified subgroups of young adults was observed. In-hospital complications were associated with increasing age. We observed no relevant differences in the median length of hospital stay. At discharge one-third of the young adults 18–50 years were free of any disability, compared to one-fifth of the adults 51–80 years of age.

**Table 2:** **Stroke care by age group**.

Characteristics	Age group (years)				
	18–50	51–80	18–30	31–40	41–50
Level of stroke care, *n* (%)					
Center	1,503 (36)	12,368 (26)	147 (46)	297 (38)	1,059 (35)
Regional	852 (21)	8,981 (19)	70 (22)	166 (21)	616 (20)
Local	1,360 (33)	18,347 (39)	77 (24)	235 (30)	1,048 (35)
Other	425 (10)	7,899 (17)	26 (8)	82 (11)	317 (10)
Admitting ward, *n* (%)					
Stroke unit	3,338 (81)	36,572 (77)	258 (81)	625 (80)	2,455 (81)
Intensive care unit	384 (9)	4,313 (9)	27 (8)	69 (9)	288 (10)
General ward	421 (10)	6,710 (14)	38 (12)	86 (11)	297 (10)
Thrombolytic therapy, *n* (%)	747 (18)	6,782 (14)	56 (18)	152 (18)	539 (18)
In-hospital complications					
Any complication, *n* (%)	299 (7)	6,335 (13)	15 (5)	52 (7)	232 (8)
Pneumonia, *n* (%)	81 (2)	2,344 (5)	2 (1)	12 (2)	67 (2)
Median length of stay in days (IQR)	7 (5, 11)	8 (5, 12)	7 (4, 11)	7 (4, 10)	7 (5, 11)
Discharge mRS score, *n* (%)					
0	1,337 (32)	8,719 (18)	140 (44)	273 (35)	924 (30)
1	1,074 (26)	10,000 (21)	78 (24)	210 (27)	786 (26)
2	803 (19)	10,430 (22)	48 (15)	143 (18)	612 (20)
3	468 (11)	8,033 (17)	25 (8)	84 (11)	359 (12)
4	251 (6)	5,210 (11)	19 (6)	32 (4)	200 (7)
5	169 (4)	3,202 (7)	10 (3)	29 (4)	130 (4)
6	38 (1)	2,001 (4)	0	9 (1)	29 (1)
Missing discharge mRS score, *n* (%)	172 (4)	2,266 (5)	12 (4)	28 (4)	132 (4)

Further subgrouping according to age and IVT revealed a clear association between admission to stroke centers and IVT (Table S2 in Supplementary Material). The proportion of IVT patients being treated in stroke centers was highest in very young stroke patients 18–30 years of age (59%), whereas the numbers in patients aged 41–50 years were comparable to those of middle age (41 and 40%, respectively). Most patients were admitted to specialized stroke units or intensive care units independent from age and/or IVT. Although in-hospital complications are likely to be underreported in our registry (any complication which required a specific therapy is required to be reported, ranging from urinary infections to cardiopulmonary resuscitation), our data reliably demonstrate an association with increasing age and IVT, in the latter, most likely related to the higher NIHSS score in the IVT groups. The same association was observed regarding the hospitalization period, which was longer in the IVT groups except for very young stroke patients 18–30 years of age, and a lower chance for being discharged functionally independent.

### Effectiveness of IVT and Mortality

Table 3 presents the numbers and percentages of patients stratified and unstratified by age with a favorable outcome at discharge defined as a mRS ≤ 1 or not worse than pre-stroke. Independent from age patients without IVT had a higher likelihood to reach a favorable outcome due to baseline imbalances between the groups, which required adjustment for several confounders for calculation of effectiveness (Tables S1 and S2 in Supplementary Material). After adjustment for patient, hospital, and treatment characteristics IVT was comparably associated with a better functional outcome in stroke patients 18–50 years of age [adjusted odds ratio (aOR) and 95% confidence interval (CI) 1.40, 1.12–1.75, *p* = 0.003] and 51–80 years of age (aOR 1.33, 95% CI 1.23–1.43). In further subgroup analysis, very young adults 18–30 years demonstrated the highest benefit nut also the largest CI (aOR 2.78, 95% CI 1.10–7.05, *p* = 0.03). Likewise, patients 31–40 years of age tended to have a more favorable outcome after IVT than patients of middle and older age, although significance was not reached (aOR 1.63, 95% CI 0.95–2.78, *p* = 0.08). Patients with 41–50 years had a similar benefit (aOR 1.33, 95% CI 1.24–1.42, *p* = 0.03). In sensitivity analyses with imputation of missing values for the NIHSS at admission and the mRS at discharge similar results to the primary analyses were observed (Table S3 in Supplementary Material).

**Table 3:** **Outcome mRS score “0–1 or not worse than pre-stroke” at discharge**.

Age group(years)	Thrombolytic therapy	No thrombolytic therapy	Adjusted OR (95%-CI)	*p*-Value
	*n* (%)	*n* (%)
18–50	340 (50)	1,920 (66)	1.40 (1.12, 1.75)	0.003
51–80	2,218 (36)	17,381 (52)	1.33 (1.23, 1.43)	<0.001
18–30	33 (62)	160 (71)	2.78 (1.10, 7.05)	0.03
31–40	70 (53)	380 (69)	1.63 (0.95, 2.78)	0.08
41–50	237 (48)	1,380 (64)	1.33 (1.02, 1.72)	0.03
Overall	2,558 (37)	19,301 (53)	1.33 (1.24, 1.42)	<0.001

*OR (binary logistic regression analysis) estimates are adjusted for pre-stroke and admission mRS scores, NIHSS score, prior stroke event, diabetes, atrial fibrillation, admitting facility and length of hospital stay. The overall estimate is additionally adjusted for age group. Numbers do not add up to group totals in Table 1 due to missing values in the outcome variable*.

*CI, confidence interval; OR, odds ratio*.

Overall mortality in patients receiving IVT was equal between the groups after adjustment for confounders (aOR 0.93, 95% CI 0.81–1.06, *p* = 0.27), and reached significance in favor of IVT after imputational inclusion of patients with missing adjustment and outcome variables (aOR 0.87, CI 0.76–0.99, *p* = 0.04, Table 4; Table S4 in Supplementary Material). In-hospital mortality was lower in young adults 18–50 years; however, a beneficial effect of IVT was not observed in this age cohort (aOR 1.32, 95% CI 0.64–2.75). Further subgroup analyses of mortality in young adults were statistically not practicable because of the low frequency of the outcome event.

**Table 4:** **Outcome in-hospital mortality**.

Age group(years)	Thrombolytic therapy	No thrombolytic therapy	Adjusted OR (95%-CI)	*p*-Value
	*n* (%)	*n* (%)
18–50	11 (2)	22 (1)	1.48 (0.68, 3.22)	0.33
51–80	388 (6)	1,291 (4)	0.92 (0.80, 1.05)	0.22
18–30	0	0		
31–40	0	7 (1)		
41–50	11 (2)	15 (1)		
Overall	399 (5)	1,313 (3)	0.93 (0.81, 1.06)	0.27

*Odds ratio estimates are adjusted for pre-stroke and admission mRS scores, NIHSS score, prior stroke event, diabetes, atrial fibrillation, admitting facility and length of hospital stay. The overall estimate is additionally adjusted for age group. Numbers do not add up to group totals in Table 1 due to missing values in the outcome variable*.

## Discussion

We present a large observational controlled analysis on the effectiveness of IVT in young adults. Approximately 51,735 ischemic stroke patients were analyzed from the BW stroke registry, of whom 7,457 (14%) underwent IVT and 4,140 (8%) were aged 18–50 years. Our results provide evidence that IVT in young adults is safe and at least equally effective as in patients of middle and older age. The highest benefit was observed in very young adults 18–30 years of age, whereas patients 41–50 years showed an equal response to treatment compared to patients 51–80 years of age. The mortality of stroke patients 51–80 years of age seemed to be lower under IVT after adjustment for confounders. However, no such association was observed in young adults 18–50 years of age. In-hospital mortality was a generally rare event in this age cohort, with a corresponding age-dependent distribution as found in Safe Implementation of Thrombolysis in Stroke – International Stroke Thrombolysis Register (SITS–ISTR) (13).

In this analysis, we observed several age-associated differences regarding baseline characteristics and stroke treatment. Very young stroke patients 18–30 years of age were in majority female, whereas males were more frequent in the other age cohorts of this study. As expected, young stroke patients suffered less cardiovascular comorbidities, which emphasizes different stroke etiologies. The functional status prior to stroke was much better compared to the elderly, and ischemic stroke severity was lower. Nevertheless, even in this quite healthy age cohort 12% experienced the at least second cerebrovascular event. Young adults had a higher chance to be treated in stroke centers providing maximum care, which are in majority university-driven hospitals. This observation might not fully reflect a specific admission pattern, since stroke centers are usually located within congested urban areas with a generally younger population. Stroke patients 18–50 years of age had a higher chance to be treated with IVT compared to patients of middle and older age. As demonstrated recently, this age cohort tends to present earlier in a hospital after stroke onset and thus might have a higher chance to be eligible for therapy (14). Moreover, a higher chance of young adults to receive IVT might be associated with the level of stroke care of the admitting hospitals. Stroke centers have a generally higher IVT rate compared to local or regional stroke units or hospitals without a stroke unit (15).

Despite to ischemic stroke patients of middle and older age data on IVT in young adults are still scarce: the Third International Stroke Trial (IST-3) reported detailed information on age and enrolled 49 patients 18–50 years treated with IVT compared to 68 age-matched controls. A subgroup analysis of this specific cohort was not published so far (16). Further data on IVT in young adults ≤50 years of age from randomized controlled trials are not available. Observational studies with at the most 63 young adults receiving IVT reported a good benefit compared to age-matched untreated controls (17–19). The SITS–ISTR analyzed 3,246 patients 18–50 years with IVT compared to 24,425 patients aged 51–80 years. According to this data, IVT is save in young stroke patients and they appear to benefit more compared to patients of middle and older age (13). However, since SITS–ISTR collects data only from patients treated with IVT a comparison to untreated age-matched controls was not possible. To overcome this limitation, SITS–ISTR was compared to the virtual international stroke trials archive (VISTA), whose patients served as an untreated control group. A combined analysis stratified into 10-year age cohorts proved effectiveness of IVT in patients aged 41–50 years. Data on patients aged 31–40 years showed a trend toward favorable outcome, whereas data on patients 21–30 years were clearly underpowered to draw robust conclusions (20).

Several limitations of our analysis have to be addressed. The main restriction of the BW stroke registry is a missing 3-month outcome parameter. Nevertheless, the authors regard the mRS at discharge as a sufficient outcome parameter, since Ovbiagele et al. demonstrated that the short-term mRS at discharge can serve as a good proxy for long-term outcome (21). Second, our study suffered a relevant proportion of missing NIHSS scores at admission and mRS scores at discharge. To avoid a substantial selection bias and thus overinterpretation of our results, a conservative procedure with imputation of missing parameters was performed under assumption of no treatment effect and the results were comparable to our primary analysis. Third, despite to careful adjustment for several confounding factors unreported confounders could have biased our results and the conclusion of our study. However, a randomized trial with adequate power to avoid unmeasured confounders is extremely difficult to organize. Fourth, no information on stroke etiology beyond the ICD 10 diagnosis was available, which makes an evaluation of specific stroke subtypes and their response to IV thrombolysis impossible. Prefasi et al. reported a lesser probability of favorable outcome in patients under 55 years of age with artery dissection (19).

## Conclusion

We present data from a large and consecutive hospital-based stroke registry located in central Europe. Out of 51,735 ischemic stroke patients included into our study, 7,457 (14%) underwent IVT and 4,140 (8%) were aged 18–50 years. Age-stratified analyses were performed using binary logistic regression models adjusted for comorbidities, stroke severity, and procedural parameters. Our analysis indicates a comparable effectiveness of IVT in young adults 18–50 years of age to patients of middle and older age. Further age-stratified subgrouping suggests that very young stroke patients 18–30 years of age might have the highest benefit.

## Conflict of Interest Statement

All authors declare: no support from any organization for the submitted work. Christoph Gumbinger holds a scholarship from the “Nachwuchsakademie Versorgungsforschung” (a health service research body) for a programme in Baden-Wuerttemberg. Rolf Kern has received speaker’s honoraria from Boehringer-Ingelheim that where unrelated to this study, Pfizer and Philips Healthcare. Peter A. Ringleb has received lecture fees and travel compensation from Boehringer-Ingelheim that where unrelated to this study, Ferrer, Paion, Bayer, and Sanofi. Werner Hacke reported honoraria from Johnson & Johnson, Bayer and advisory board fees from Boehringer-Ingelheim that where unrelated to this study. Michael G. Hennerici has received research grant support from Pfizer.

## Stroke Working Group of Baden-Wuerttemberg

Stroke Working Group of the Federal State of Baden-Wuerttemberg, Stuttgart, Germany: K. Schoser (Klinikum Tuttlingen, Germany), M. Daffertshofer (Department of Neurology, Stadtklinik Baden-Baden, Germany), C. Diehm (Department of Internal/Vascular Medicine, Max-Grundig-Klinik, Bühl, Germany), S. Neumaier (Medical Services of Health Insurance Freiburg, Germany), B. Müller-Jenz (Medical Services of Health Insurance Stuttgart, Germany), E Drewitz (Department of Neurology, Klinikum Stuttgart, Germany).
