# Supplementary material for: Intravenous Thrombolysis is Effective in Young Adults: Results from the Baden-Wuerttemberg Stroke Registry
Source: Front Neurol. 2015 Nov 4;6:229. doi: 10.3389/fneur.2015.00229 (PMC4631948; doi:10.3389/fneur.2015.00229)
Supplement: Supplementary file 1 [file table_1.docx]

| **Table S1.** Patient characteristics stratified by age group and IVT | | | | | | | | | | | | |
| --- | --- | --- | --- | --- | --- | --- | --- | --- | --- | --- | --- | --- |
| **Age group** | | **18-50 years** | | **51-80 years** | |  | **18-30 years** | | **31-40 years** | | **41-50 years** | |
| **IVT** | | **yes** | **no** | **yes** | **no** |  | **yes** | **no** | **yes** | **no** | **yes** | **no** |
| Patients, n (%) | | 737 (18) | 3403 (82) | 6782 (14) | 40813 (14) |  | 56 (18) | 264 (82) | 142 (18) | 638 (82) | 539 (18) | 2501 (82) |
| Female sex, n (%) | | 276 (37) | 1383 (41) | 2716 (40) | 16678 (41) |  | 28 (50) | 153 (58) | 63 (44) | 284 (45) | 185 (34) | 946 (38) |
| Pre-stroke mRS score, n (%) | |  |  |  |  |  |  |  |  |  |  |  |
|  | 0 | 686 (93) | 2996 (88) | 5239 (77) | 27054 (66) |  | 51 (91) | 251 (95) | 137 (97) | 575 (90) | 498 (92) | 2170 (87) |
|  | 1 | 28 (4) | 211 (6) | 707 (10) | 5233 (13) |  | 4 (7) | 6 (2) | 4 (3) | 37 (6) | 20 (4) | 168 (7) |
|  | 2 | 14 (2) | 111 (3) | 418 (6) | 4268 (11) |  | 0 | 4 (2) | 0 | 19 (3) | 14 (3) | 88 (4) |
|  | 3 | 5 (1) | 65 (2) | 299 (4) | 2786 (7) |  | 1 (2) | 2 (1) | 1 81) | 5 (1) | 3 (1) | 58 (2) |
|  | 4 | 4 (1) | 18 (1) | 101 (2) | 1191 (3) |  | 0 | 1 (0) | 0 | 2 (0) | 4 (1) | 15 (1) |
|  | 5 | 0 | 2 (0) | 18 (0) | 281 (1) |  | 0 | 0 | 0 | 0 | 0 | 2 (0) |
| NIHSS, median (IQR) | | 7 (4, 11) | 2 (1, 4) | 8 (5, 14) | 3 (2, 7) |  | 5 (3, 10) | 2 (1, 4) | 7 (4, 13) | 2 (1, 4) | 7 (4, 11) | 2 (1, 5) |
| missing NIHSS, n (%) | | 14 (2) | 371 (11) | 121 (2) | 5739 (14) |  | 0 | 32 (12) | 3 (2) | 70 (11) | 11 (2) | 269 (11) |
| Comorbidities, n (%) | |  |  |  |  |  |  |  |  |  |  |  |
|  | Arterial hypertension^a^ | 194 (39) | 929 (44) | 3930 (84) | 20873 (84) |  | 6 (15) | 16 (9) | 17 (19) | 114 (30) | 171 (47) | 799 (51) |
|  | Hypercholesterolemia^a^ | 144 (29) | 766 (36) | 2476 (53) | 13779 (56) |  | 5 (12) | 24 (13) | 14 (15) | 97 (25) | 125 (35) | 645 (42) |
|  | Atrial fibrillation | 41 (6) | 126 (4) | 1982 (29) | 9715 (24) |  | 4 (7) | 1 (0) | 4 (3) | 18 (3) | 33 (6) | 107 (4) |
|  | Diabetes mellitus | 48 (7) | 359 (11) | 1653 (24) | 12348 (30) |  | 0 | 9 (3) | 5 (4) | 47 (7) | 43 (8) | 303 (12) |
| Prior stroke event, n (%) | | 489 (12) | 65 (9) | 1139 (17) | 10970 (27) |  | 4 (7) | 20 (8) | 7 (5) | 65 (10) | 54 (10) | 339 (14) |
| ^a^ Information was not routinely documented over the entire study period and is therefore missing for N=19703 patients. Abbreviations: IVT, intravenous thrombolysis; IQR, interquartile range; mRS, modified Rankin Scale; NIHSS, National Institutes of Health stroke scale. | | | | | | | | | | | | |
