# Supplementary material for: Intravenous Thrombolysis is Effective in Young Adults: Results from the Baden-Wuerttemberg Stroke Registry
Source: Front Neurol. 2015 Nov 4;6:229. doi: 10.3389/fneur.2015.00229 (PMC4631948; doi:10.3389/fneur.2015.00229)
Supplement: Supplementary file 2 [file table_2.docx]

| **Table S2.** Stroke care stratified by age group and IVT | | | | | | | | | | | | |
| --- | --- | --- | --- | --- | --- | --- | --- | --- | --- | --- | --- | --- |
| **Age group** | | **18-50 years** | | **51-80 years** | |  | **18-30 years** | | **31-40 years** | | **41-50 years** | |
| **IVT** | | **yes** | **no** | **yes** | **no** |  | **yes** | **no** | **yes** | **no** | **yes** | **no** |
| Level of stroke care, n (%) | |  |  |  |  |  |  |  |  |  |  |  |
|  | Center | 326 (44) | 1177 (35) | 2690 (40) | 9678 (24) |  | 33 (59) | 114 (43) | 70 (49) | 227 (36) | 223 (41) | 836 (33) |
|  | Regional | 137 (19) | 715 (21) | 1247 (18) | 7734 (19) |  | 13 (23) | 57 (22) | 24 (17) | 142 (22) | 100 (19) | 516 821) |
|  | Local | 221 (30) | 1139 (34) | 2250 (33) | 16097 (39) |  | 9 (16) | 68 (26) | 40 (28) | 195 (31) | 172 (32) | 876 (35) |
|  | Other | 53 (7) | 372 (11) | 595 (9) | 7304 (18) |  | 1 (2) | 25 (10) | 8 (6) | 74 (12) | 44 (8) | 273 811) |
| Admitting ward, n (%) | |  |  |  |  |  |  |  |  |  |  |  |
|  | Stroke unit | 596 (81) | 2742 (81) | 5566 (82) | 31006 (76) |  | 51 (91) | 207 (78) | 115 (81) | 510 (80) | 430 (80) | 2025 (81) |
|  | Intensive care unit | 134 (18) | 247 (7) | 1141 (17) | 3172 (8) |  | 5 (9) | 19 (7) | 25 (18) | 44 (7) | 104 (19) | 184 (7) |
|  | General ward | 7 (1) | 414 (12) | 75 (1) | 6635 (16) |  | 0 | 38 (14) | 2 (1) | 84 (13) | 5 (1) | 292 (12) |
| In-hospital complications, n (%) | | 79 (11) | 220 (7) | 1448 (21) | 4887 (12) |  | 3 (5) | 12 (5) | 15 (11) | 37 (6) | 61 (11) | 171 (7) |
| Median length of stay in days (IQR) | | 8 (5, 11) | 7 (4, 10) | 9 (6, 14) | 8 (5, 12) |  | 6 (3, 11) | 7 (5, 11) | 8 (5, 12) | 6 (4, 10) | 8 (5, 11) | 7 (4, 11) |
| Discharge mRS score, n (%) | |  |  |  |  |  |  |  |  |  |  |  |
|  | 0 | 172 (25) | 1158 (35) | 951 (15) | 7732 (20) |  | 13 (25) | 127 (50) | 35 (26) | 237 (38) | 124 (25) | 794 (33) |
|  | 1 | 167 (24) | 886 (27) | 1048 (17) | 8790 (23) |  | 19 (36) | 57 (22) | 35 (26) | 170 (28) | 113 (22) | 659 (27) |
|  | 2 | 130 (19) | 652 820) | 1201 (19) | 8894 (23) |  | 9 (17) | 38 (15) | 25 (19) | 116 (19) | 96 (19) | 498 (21) |
|  | 3 | 106 (15) | 326 (10) | 1167 (18) | 6326 (16) |  | 8 (15) | 14 (6) | 22 (16) | 57 (9) | 76 (15) | 255 (11) |
|  | 4 | 62 (9) | 153 (5) | 924 (15) | 3657 (9) |  | 3 (6) | 12 (5) | 7 (5) | 21 (3) | 52 (10) | 120 (5) |
|  | 5 | 43 (6) | 75 (2) | 649 (10) | 1989 (5) |  | 1 (2) | 7 (3) | 9 (7) | 9 82) | 33 (7) | 59 (3) |
|  | 6 | 12 (2) | 26 (1) | 400 (6) | 1601 (4) |  | 0 | 0 | 1 (1) | 8 (1) | 11 (2) | 18 (1) |
| missing discharge mRS score, n (%) | | 45 (6) | 127 (4) | 442 (7) | 1824 (5) |  | 3 (5) | 9 (3) | 8 (6) | 20 (3) | 34 (6) | 98 (4) |
