# Supplementary material for: Intravenous Thrombolysis is Effective in Young Adults: Results from the Baden-Wuerttemberg Stroke Registry
Source: Front Neurol. 2015 Nov 4;6:229. doi: 10.3389/fneur.2015.00229 (PMC4631948; doi:10.3389/fneur.2015.00229)
Supplement: Supplementary file 3 [file table_3.docx]

| **Table S3.** Outcome mRS score 0-1 or no worse than pre-stroke at discharge (binary logistic regression analysis with imputation of missing variables). | | | | | |
| --- | --- | --- | --- | --- | --- |
| **Age group** | **Thrombolytic therapy** |  | **No thrombolytic therapy** | **Adjusted OR  (95%-CI)** | **P value** |
|  | **n (%)** |  | **n (%)** |  |  |
| 18-50 years | 348 (47) |  | 2171 (64) | 1.45 (1.17, 1.79) | <0.001 |
| 51-80 years | 2264 (33) |  | 20567 (50) | 1.29 (1.21, 1.38) | <0.001 |
|  |  |  |  |  |  |
| 18-30 years | 33(59) |  | 187 (71) | 2.83 (1.13, 7.09) | 0.03 |
| 31-40 years | 71 (50) |  | 429 (67) | 1.52 (0.92, 2.52) | 0.1 |
| 41-50 years | 244 (45) |  | 1555 (62) | 1.39 (1.09, 1.79) | 0.01 |
| Overall | 2612 (35) |  | 22738 (51) | 1.30 (1.22, 1.39) | <0.001 |
| OR estimates are adjusted for pre-stroke and admission mRS scores, NIHSS score, prior stroke event, diabetes, atrial fibrillation, admitting facility and length of hospital stay. The overall estimate is additionally adjusted for age group. Numbers do not add up to group totals in Table 1 due to missing values in the outcome variable. | | | | | |
