# Supplementary material for: Intravenous Thrombolysis is Effective in Young Adults: Results from the Baden-Wuerttemberg Stroke Registry
Source: Front Neurol. 2015 Nov 4;6:229. doi: 10.3389/fneur.2015.00229 (PMC4631948; doi:10.3389/fneur.2015.00229)
Supplement: Supplementary file 4 [file table_4.docx]

| **Table S4.** Outcome in-hospital mortality (binary logistic regression analysis with imputation of missing endpoint variables). | | | | | |
| --- | --- | --- | --- | --- | --- |
| **Age group** | **Thrombolytic therapy** |  | **No thrombolytic therapy** | **Adjusted OR  (95%-CI)** | **P value** |
|  | **n (%)** |  | **n (%)** |  |  |
| 18-50 years | 12 (2) |  | 26 (1) | 1.32 (0.64, 2.75) | 0.45 |
| 51-80 years | 400 (6) |  | 1601 (4) | 0.86 (0.76, 0.99) | 0.03 |
|  |  |  |  |  |  |
| 18-30 years | 0 |  | 0 |  |  |
| 31-40 years | 1 (1) |  | 8 (1) |  |  |
| 41-50 years | 11 (2) |  | 18 (1) |  |  |
| Overall | 412 (5) |  | 1627 (4) | 0.87 (0.76, 0.99) | 0.04 |
| OR estimates are adjusted for pre-stroke and admission mRS scores, NIHSS score, prior stroke event, diabetes, atrial fibrillation, admitting facility and length of hospital stay. The overall estimate is additionally adjusted for age group. Numbers do not add up to group totals in Table 1 due to missing values in the outcome variable. | | | | | |
